# Forked-Crossing Metasurface for Multi-Band Polarization Conversion with Distinct Bandwidths

**DOI:** 10.3390/mi16101100

**Published:** 2025-09-28

**Authors:** Pengpeng Zhang, Yifei Zhang, Ziang Li, Rui Yang

**Affiliations:** 1 China Airborne Missile Academy, Luoyang 471009, China; pengpengzhang2025@163.com; 2 National Key Laboratory of Air-Based Information Perception and Fusion, Luoyang 471009, China; 3 National Key Laboratory of Radar Detection and Sensing, School of Electronic Engineering, Xidian University, Xi’an 710071, China; 24021211782@stu.xidian.edu.cn

**Keywords:** multi-band, metasurface polarization converter, distinct bandwidths

## Abstract

This study presents a reflective and highly efficient multi-band metasurface polarization converter based on a forked-crossing patch array. Both simulation and experimental results reveal that such a metasurface achieves polarization conversion ratio (PCR) exceeding 90% over five frequency bands of 4.71–5.44 GHz, 7.26–9.55 GHz, 11.62–12.6 GHz, 13.33–13.46 GHz, and 15.61–15.62 GHz with high conversion efficiency realized at five distinct resonances. The quality-factor (Q-factor) analysis of each band reveals a hybrid behavior. More specifically, the first and second bands exhibit relatively low Q factors of approximately 6.95 and 3.67, indicating wideband polarization conversion capability. The third band has a moderate Q factor of 12.35, while the fourth and fifth bands show high-Q resonances with Q factors of 103.04 and 1561.5, respectively, indicating sharp and selective frequency responses. This combination of wideband and high-Q narrowband responses makes the proposed design especially suitable for complex electromagnetic scenarios, such as multifunctional radar, communication, and sensing systems, where both broad frequency coverage and precise spectral control are simultaneously required.

## 1. Introduction

A polarization converter is a device designed to manipulate the polarization state of electromagnetic (EM) waves, enabling transformation from one polarization to another. Depending on their working principles, polarization converters can realize linear-to-linear, linear-to-circular, or circular-to-linear conversions in either transmission or reflection modes. Recent advances in metasurface technology have spurred the development of novel and compact polarization conversion devices with enhanced performance and functionality [1,2,3,4,5,6]. A diverse range of metasurface-based designs have been reported, significantly broadening their application spectrum in modern electromagnetic systems. These include ultra-wideband and highly efficient cross-polarization converters [7,8,9,10,11,12], which are particularly suitable for high-capacity communications and sensing systems.

Multi-band polarization converters offer substantial advantages for practical applications [13,14,15,16,17,18,19,20,21,22]. In [18], Hossain et al. proposed a compact tri-band reflective polarization converter using square split-ring resonators integrated with metallic cross elements. In [19], Yang et al. presented a tri-band reflective polarization conversion metasurface employing two metallic rectangular ring resonators arranged on a single-layer reflective substrate. This configuration demonstrated high polarization conversion efficiency across three discrete frequency bands. Abbas et al. [20] reported an ultra-wideband reflective polarization converter composed of three anisotropic stair-shaped resonators. Their metasurface achieved efficient cross-polarization conversion over two wide frequency bands, attaining PCR exceeding 90%. In [21], Mustafa et al. designed an orthotropic metasurface reflector that achieved cross-polarization conversion in two bands, with a wideband response in the first band and a narrowband response in the second, while in [22], Han and Chen proposed a dual-band metasurface realizing efficient asymmetric transmission.

Despite these promising designs, most reported metasurfaces are restricted to a limited number of frequency bands and often fail to achieve distinct bandwidth coexistence. Integrating wideband and narrowband responses in a single metasurface remains technically challenging because multiple resonances tend to couple strongly, distort targeted bandwidths, and degrade polarization conversion efficiency. Structural complexity further complicates optimization. However, in many real-world applications such as radar and secure communication, the ability to combine wideband coverage with ultra-narrowband selectivity is highly desirable. In this work, we propose a reflective linear-to-linear cross-polarization converter that transforms an incident *y*-polarized wave into an *x*-polarized reflected wave with high efficiency. The design leverages a forked-crossing patch geometry, where the forked arms direct surface currents into distinct pathways, effectively decoupling magnetic and electric resonances to enable broadband conversion together with high-Q narrowband responses. Benefiting from this hybrid mechanism, the proposed design achieves highly efficient polarization conversion (PCR > 90%) across five distinct frequency bands: 4.71–5.44 GHz, 7.26–9.55 GHz, 11.62–12.6 GHz, 13.33–13.46 GHz, and 15.61–15.62 GHz. This highlights its novelty and potential for multifunctional applications in radar, communication, and sensing.

## 2. Modeling and Numerical Results

**Figure 1:**
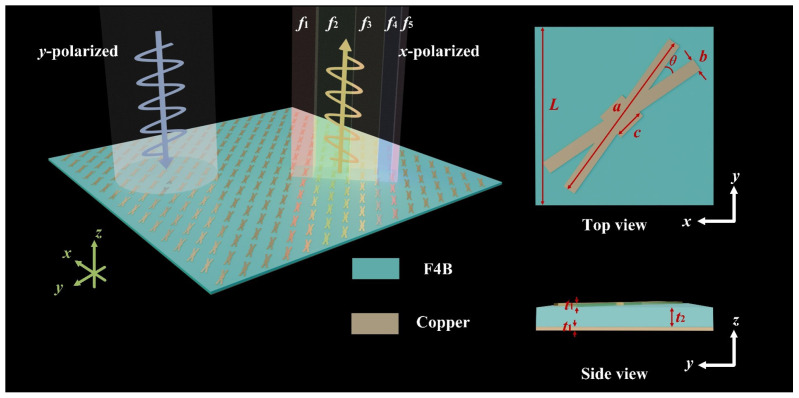
Schematic diagram of the proposed forked-crossing metasurface polarization converter.

The proposed reflective metasurface polarization converter consists of periodically arranged forked-crossing unit array, as illustrated in Figure 1. The five different colors of the reflected waves in Figure 1 represent five distinct conversion frequency bands. The polarization converter features a unit cell length of L=11 mm, with both the top and bottom surfaces constructed of copper with a thickness of $t_{1}=0.07$ mm. The dielectric layer is fabricated using F4B material, which has a dielectric constant of 4.4 and a substrate thickness of $t_{2}=4$ mm. The metasurface’s top patch is symmetrical along the substrate diagonal. Further structural parameters are a=11.69 mm, b=2 mm, c=1.9 mm, θ=20°. Through the optimization of the diagonal placement and rotation angle, the cross-polarization conversion effect is enhanced.

### 2.1. Simulation Setup

The polarization conversion properties of the proposed metasurface were numerically investigated using the frequency-domain solver of CST Microwave Studio. Periodic boundary conditions were applied along the *x* and *y* directions to emulate an infinite array of unit cells, while open boundary conditions were set along the propagation (*z*) direction. The structure was excited through Floquet ports, which enable rigorous plane-wave expansion for periodic structures. A normally incident *y*-polarized fundamental Floquet mode was employed, corresponding to normal incidence in free space. The input port power was set to 1 W, and the reflected Floquet modes were used to extract the co- and cross-polarized reflection coefficients, from which the polarization conversion ratio (PCR) was calculated. An adaptive mesh refinement strategy was employed, with a mesh density corresponding to approximately λ/20 at the highest frequency of 16 GHz. To accurately capture fine geometric details, especially around metallic edges, the minimum mesh size was restricted to 0.55 mm. The convergence criterion was defined as a change in S parameters below −40 dB between consecutive iterations, ensuring numerical stability and accuracy.

**Figure 2:**
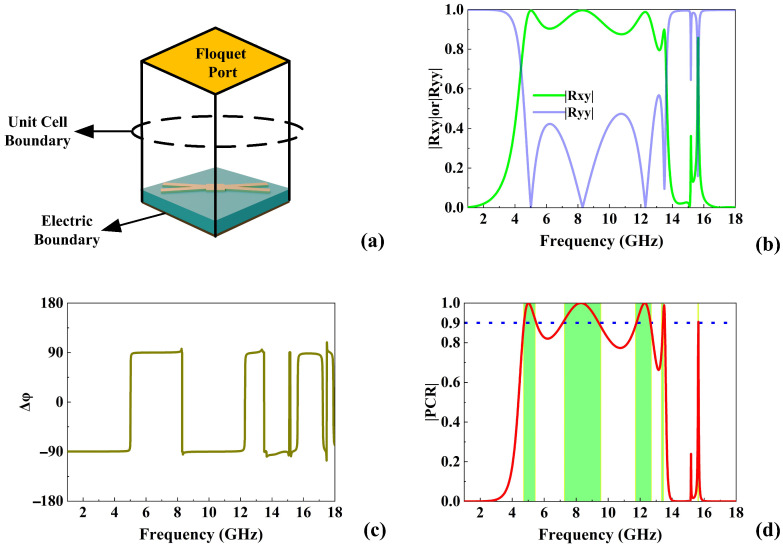
(**a**) Floquet mode analysis; spectra of (**b**) reflection coefficients, (**c**) Δφ, and (**d**) PCR.

Figure 2a demonstrates the Floquet mode analysis of the proposed forked-crossing metasurface polarization converter. A *y*-polarized electromagnetic wave is incident normally on the top of the structure. The components of the electric field of the incident wave and the reflected wave can be related to each other by the Jones matrix:

(1) $$\left(\begin{array}{c} E_{xr}\\ E_{yr} \end{array}\right)=\left(\begin{array}{cc} R_{xx} & R_{xy}\\ R_{yx} & R_{yy} \end{array}\right)\left(\begin{array}{c} E_{xi}\\ E_{yi} \end{array}\right)$$

where $R_{xy}$ and $R_{yy}$ are the cross-polarization reflection coefficient from *y*-polarized incidence to *x*-polarized reflection and the co-polarization reflection coefficient from *y*-polarized incidence to *y*-polarized reflection, respectively. To better evaluate the polarization conversion performance, PCR is introduced and calculated as follows:

(2) $$PCR=\frac{{\left|R_{xy}\right|}^{2}}{{\left|R_{xy}\right|}^{2}+{\left|R_{yy}\right|}^{2}}$$

A higher PCR indicates better performance of the polarization converter.

The spectra of cross-polarization ($R_{xy}$) and co-polarization ($R_{yy}$) reflection coefficients are shown in Figure 2b. The spectra of phase difference (Δφ) are depicted in Figure 2c, defined as the phase difference between the incident wave and the reflected wave, and the calculation formula is as follows:

(3) $$\Delta \varphi =\varphi_{reflected}-\varphi_{incident}$$

It can be observed that in the frequency ranges of 4.71–5.44 GHz, 7.26–9.55 GHz, 11.62–12.6 GHz, 13.33–13.46 GHz, and 15.61–15.62 GHz, $R_{xy}$ is greater than 0.8, and $R_{yy}$ is less than 0.2. The phase difference in these frequency ranges is equal to ±90°, indicating that the *y*-polarized incident wave energy is converted to the *x*-polarized reflected wave. As shown in Figure 2d, in these frequency ranges, the PCR exceeds 0.9, and the structure has cross-polarization conversion performance in multi-band frequency ranges.

### 2.2. Analysis of Resonance Mechanism

**Figure 3:**
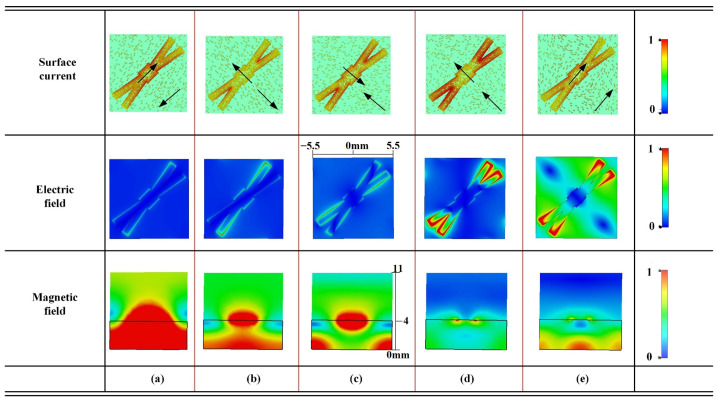
Field distributions of the proposed metasurface at five resonant frequencies: (**a**) *f* = 5 GHz, (**b**) *f* = 8.36 GHz, (**c**) *f* = 12.2 GHz, (**d**) *f* = 13.41 GHz, and (**e**) *f* = 15.618 GHz.

To gain deeper insight into the physical mechanisms of polarization conversion, Figure 3 presents the simulated surface current distributions, electric field distributions, and magnetic field patterns at five distinct resonant frequencies: 5 GHz, 8.36 GHz, 12.2 GHz, 13.41 GHz, and 15.618 GHz. The surface current distributions reveal the direction relationship of induced currents on the top metallic patch and the ground plane, serving as indicators for classifying magnetic or electric resonances. More importantly, the corresponding electric and magnetic field distributions offer intuitive, spatially resolved evidence of the underlying electromagnetic modal behavior.

At 5 GHz, 8.36 GHz, and 12.2 GHz, the surface currents on the top patch and ground plane flow in opposite directions, forming loop-like configurations characteristic of magnetic resonances. The electric fields exhibit approximately antisymmetric patterns around the structural gaps and edges, indicating voltage buildup along inductive paths. The associated magnetic fields are broadly distributed across the substrate, implying weak electromagnetic confinement. These features collectively correspond to low-Q, wideband resonance behavior. In contrast, at 13.41 GHz and 15.618 GHz, the surface currents on both conducting layers flow in the same direction, consistent with electric resonances. The electric fields are strongly localized at specific structural discontinuities of the splits and the corners. The magnetic field distributions, especially in side views, exhibit tightly confined energy beneath the patch, indicating strong field localization and a high-Q, narrowband resonance response. Moreover, the magnetic field distributions in Figure 3 exhibit noticeable asymmetries. These asymmetries arise from the forked-crossing geometry, where the diagonal arrangement of the arms introduces uneven current paths and consequently non-uniform magnetic loops. Such field asymmetry is not a numerical artifact but an intrinsic feature of the structure. It effectively reduces modal coupling between adjacent resonances and facilitates the coexistence of wideband magnetic modes and narrowband electric modes within the same metasurface.

To quantify the field localization at each resonance, we introduce the field confinement factor (FCF), defined as

(4) $$FCF=\frac{\int_{V_{core}}u\left(r\right)dV}{\int_{V_{total}}u\left(r\right)dV}$$

where $u\left(r\right)=\frac{1}{2}\left(\varepsilon {\left|E\right|}^{2}+\mu {\left|H\right|}^{2}\right)$ denotes the electromagnetic energy density. Here, $V_{total}$ refers to the unit cell volume bounded by the periodic boundaries in the *x*–*y* plane and the open boundaries along *z*, while $V_{core}$ is the sub-volume where the energy density exceeds 20% of its maximum value at the resonance frequency. This definition allows a consistent comparison of field confinement across different modes.

Table 1 quantitatively summarizes the resonance characteristics by combining Q factor, FCF, and mode classification. A clear correlation emerges: magnetic resonances feature weak confinement and low Q factors, resulting in wideband responses. In contrast, electric resonances exhibit strong confinement and significantly higher Q factors, leading to narrowband and ultra-narrowband responses. This quantitative evidence demonstrates that stronger field confinement directly contributes to higher Q factors, consistent with the current distributions observed in Figure 3.

These observations highlight a hybrid resonance mechanism operating in the metasurface. Magnetic resonant modes facilitate wideband polarization conversion due to lower field confinement and higher radiation leakage, while electric modes support narrowband responses through enhanced field localization and lower radiative losses. The coexistence and interplay of these distinct modal behaviors allow the metasurface to deliver polarization conversion functionalities across multiple frequency bands, each with distinct bandwidths.

This hybrid mechanism also clarifies how the forked-crossing geometry overcomes the technical challenges of integrating wideband and narrowband responses. Without proper current guidance, the coexistence of distinct modes could suffer from strong intermodal coupling and reduced efficiency. As shown in Figure 3 and quantified in Table 1, the forked arms steer magnetic-type and electric-type currents into separate regions, thereby isolating wideband and narrowband resonances. This spatial separation suppresses unwanted energy exchange and ensures that both types of responses can coexist with high polarization conversion efficiency.

### 2.3. Parameter Optimization and Angular Stability

**Figure 4:**
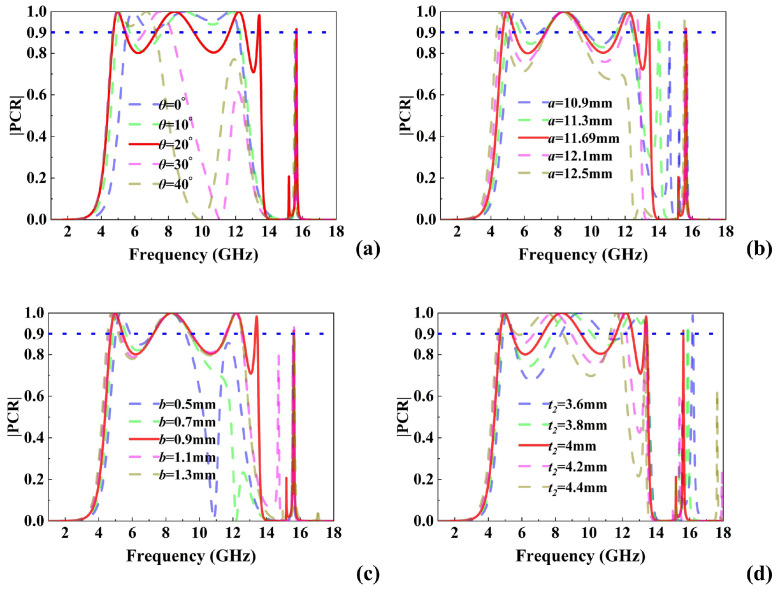
Structural parameter analysis. PCR varies with (**a**) θ, (**b**) *a*, (**c**) *b*. (**d**) $t_{2}$.

To investigate the influence of structural parameter variations on the polarization conversion performance of the metasurface, the effects of the included angle θ, the length *a* and width *b* of the top metallic patch, and the dielectric substrate thickness $t_{2}$ were analyzed. As illustrated in Figure 4, variations in these parameters primarily result in the shifting, emergence, or disappearance of certain resonance points, thereby impacting the polarization conversion efficiency and multi-band characteristics. Figure 4a demonstrates that changes in θ primarily induce frequency shifts and the appearance or disappearance of resonance modes, significantly influencing both conversion efficiency and multi-band characteristics. Specifically, increasing θ shifts the second resonance point toward higher frequencies and promotes the emergence of the third and fourth resonances, thereby weakening the coupling effect while enhancing the multi-band performance. However, once a critical threshold is exceeded, further increases in θ lead to a high-frequency shift in the first resonance and the disappearance of the fourth and fifth resonance modes, thereby degrading the multi-band performance. As shown in Figure 4b, variations in a mainly affect the first, third, and fourth resonance points. Increasing a results in a low-frequency shift in the first resonance, a high-frequency shift in the third, and the emergence of the fourth, collectively improving the multi-band characteristics. However, beyond a critical value of *a*, both the third and fourth resonance points vanish. Figure 4c reveals that changes in *b* primarily influence the first, third, and fourth resonance modes. An increase in *b* causes the first resonance to shift toward lower frequencies, while the third and fourth resonances gradually emerge, enhancing the multi-band response. Nevertheless, surpassing a threshold value of *b* leads to the disappearance of the fourth resonance. Lastly, Figure 4d shows that variations in the dielectric thickness $t_{2}$ primarily affect the second, third, and fifth resonance points. Increasing $t_{2}$ results in low-frequency shifts in the second and third resonances, thereby enhancing both the multi-band characteristics and Q factor. However, exceeding the critical value of $t_{2}$ causes the fifth resonance point to disappear.

**Figure 5:**
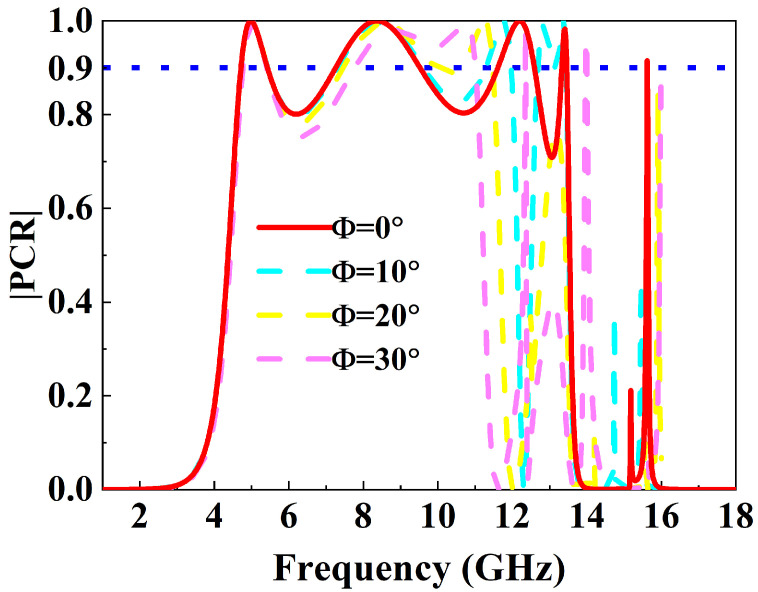
PCR spectrum as a function of incident angle.

Furthermore, to evaluate practical applicability, the PCR was simulated under oblique incidence for angles from 0° to 30°, as shown in Figure 5. The results indicate that the first two broadband resonances (4.71–5.44 GHz and 7.26–9.55 GHz) maintain PCR above 85% and exhibit excellent angular stability, whereas the narrowband resonances (11.62–12.6, 13.33–13.46, and 15.61–15.62 GHz) are highly sensitive to incidence angle, showing significant frequency shifts and rapid PCR degradation. These findings demonstrate that the metasurface offers robust angular stability for wideband C- and X-band applications, while narrowband Ku-band usage requires stricter alignment or array-level optimization.

## 3. Experimental Verification and Comparisons

### 3.1. Fabrication and Calibration

The metasurface sample was fabricated using the etching method, a standard approach for printed circuit board (PCB) patterning. The process followed the industrial standard IPC-600F Class II, which specifies requirements for conductor edge quality, copper adhesion, defect tolerance, and substrate flatness. Although the manufacturer did not provide additional process details such as copper surface uniformity or substrate polishing procedures, compliance with IPC-600F-II ensured that the fabricated metasurface met the general quality requirements for high-frequency electromagnetic applications. The dielectric substrate used in this work was F4B ($\varepsilon_{r}=4.4$, loss tangent of tanδ=0.001), with a nominal thickness of 4 mm and copper cladding of 0.07 mm. To account for possible deviations in material properties introduced during fabrication, these parameters were employed in the lossy simulations, which enabled a more accurate comparison between numerical and experimental results.

For the experimental verification, a thorough calibration procedure was conducted prior to measurement. A vector network analyzer was calibrated using the short-open-load-through method to eliminate systematic errors. In addition, the transmitting and receiving horn antennas were carefully aligned to minimize angular misalignment and spurious coupling. These calibration steps ensured that the measured PCR accurately reflected the intrinsic performance of the fabricated metasurface.

### 3.2. Experimental Setup and Results

**Figure 6:**
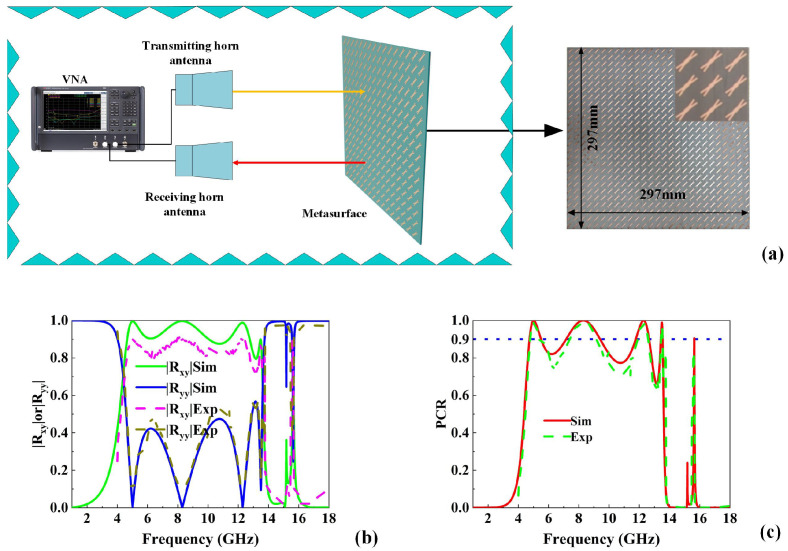
(**a**) Schematic diagram of the experiment setup and the testing sample; comparison between the simulation and the experiment results of (**b**) reflection coefficients and (**c**) PCR.

Finally, we fabricated the proposed metasurface and carried out the experiments to verify our proposed design as shown in Figure 6. In the fabrication, the F4B circuit board ($\varepsilon_{r}=4.4$, loss tangent of tanδ=0.001) was chosen as the dielectric substrate for the metasurface. As shown in Figure 6a, the test sample consisted of a 27 × 27 array of unit cells on a 297 mm × 297 mm substrate. The experiment setup contained a vector network analyzer and two linearly polarized horn antennas. A wideband horn antenna emitted the *y*-polarized wave, while another captured the reflected signal. As observed inFigure 6b,c, the proposed metasurface achieves PCR exceeding 90% within the frequency bands of 4.8–5.4 GHz, 7.3–9.35 GHz, 11.84–12.6 GHz, and 13.45–13.5 GHz, and PCR exceeding 80% in the band of 15.615–15.618 GHz. Compared with the simulations, the measured PCR exhibits a slight decrease, and the conversion bandwidths are narrower. This is mainly attributed to the fabrication tolerances and material losses. However, the overall performance of this metasurface fully demonstrates its ability to produce polarization conversion with distinct bandwidths in multiple frequency bands that agrees quite well with our design.

### 3.3. Impact of Material Losses

**Figure 7:**
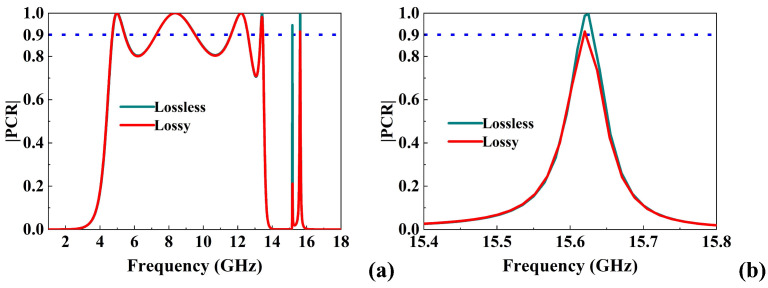
(**a**) Comparison of simulated PCR between the lossless case and the realistic lossy case, (**b**) microscopic enlarged view of the fifth conversion frequency band.

All full-wave simulations presented in this work were performed using realistic lossy parameters, including copper conductivity ($\sigma =5.8\times 10^{7}$ S/m) and the dielectric loss tangent of the F4B substrate (tanδ=0.001). To highlight the effect of losses, Figure 7a compares the PCR for the lossless case with PEC conductor and F4B (tanδ=0) and the realistic lossy case, and Figure 7b is the microscopic enlarged view of the fifth conversion frequency band. As shown, the lossless model predicts nearly ideal PCR (>0.95) across all operating bands. In contrast, the lossy simulation reproduces the degradation of PCR and the narrowing of bandwidth observed in measurements, especially in the high-Q narrowbands at 13–16 GHz. The comparison confirms that finite copper conductivity and dielectric dissipation are the main contributors to the reduced PCR performance. This agreement also demonstrates that the inclusion of lossy material parameters is essential for accurately predicting experimental results.

### 3.4. Comparison with Previous Works

As summarized in Table 2, this comparison highlights that most designs either realize broadband or multi-band operation but rarely integrate broadband and ultra-narrowband characteristics within a single metasurface. For instance, Ren et al. [5] achieved ultra-wideband polarization conversion but did not realize the coexistence of wideband and ultra-narrowband response. The tri-band metasurface proposed by Hossain et al. [18] and Yang et al. [19] and the dual wideband converter reported by Abbas et al. [20] successfully realized multi-band cross-polarization conversion. However, the number of effective bands in both cases remained limited. Other studies, such as those in [21], primarily focused on two-band performance, exhibiting restricted polarization conversion efficiency and limited spectral coverage. In contrast, the proposed forked-crossing metasurface polarization converter achieves efficient polarization conversion across five distinct frequency bands: 4.71–5.44 GHz, 7.26–9.55 GHz, 11.62–12.6 GHz, 13.33–13.46 GHz, and 15.61–15.62 GHz. This performance not only surpasses the state of the art in terms of the number of operational bands but also demonstrates the ability to integrate different bandwidth characteristics within a single structure. Such a combination significantly enhances the functional diversity and spectral adaptability of the device, rendering it particularly suitable for complex electromagnetic environments and multi-standard communication systems.

## 4. Conclusions

This study presented a reflective forked-crossing metasurface that achieved multi-band polarization conversion with distinct bandwidth characteristics. Simulations and experiments confirmed five operating bands (4.71–5.44, 7.26–9.55, 11.62–12.6, 13.33–13.46, and 15.61–15.62 GHz), with the PCR exceeding 90% in most cases. Modal analysis indicated that wideband, angle-stable responses originates from low-Q magnetic resonances, while high-Q electric resonances provide narrowband, high-selectivity operation. Comparison between lossless and lossy cases showed that material absorption was mainly responsible for the PCR reduction and bandwidth narrowing observed in experiments. Angular dependence up to 30° incidence further demonstrates that wideband modes remain stable, whereas narrowband modes are more sensitive to incidence angle. The five operating bands correspond to practical radar and communication frequencies: C band (4.71–5.44 GHz) for weather radar and satellite links, X- (7.26–9.55 GHz) for radar and SAR applications, Ku band (11.62–12.6 and 13.33–13.46 GHz) for satellite broadcasting and remote sensing, and ultra-narrowband resonance (15.61–15.62 GHz) suitable for precision tracking or target locking. These mappings underline the engineering relevance of the design. The proposed metasurface combines wideband angular stability with narrowband spectral selectivity in a compact form, making it a promising candidate for multifunctional radar and communication systems.

## Figures and Tables

**Table 1:** Resonance characteristics of the proposed metasurface.

Resonance (GHz)	Mode Type	FCF	Q Factor	Bandwidth Type
5.0	Magnetic	0.022	6.95	Wide
8.36	Magnetic	0.007	3.67	Wide
12.2	Magnetic	0.037	12.35	Wide
13.41	Electric	0.070	103.04	Narrow
15.618	Electric	0.081	1561.5	Ultra-narrow

**Table 2:** Comparison with other polarization converters.

Reference	Operating Bandwidth (GHz)	Bandwidth Type	Max/Min Relative Bandwidth (%)	Q Factor	Structural Complexity	Reported Applications
[5]	7.1–22.3	Ultra-wideband	103.4	0.97	single layer	6G communications, radar imaging, anti-interference measures, and electromagnetic stealth
[18]	5.35–5.69; 7.60–8.76; 12.41–13.96	Wideband and narrowband coexistence	14.2/6.2	16.24, 7.05, 8.51	single layer	Radar, satellite communications, and wireless communications
[19]	9.1–12.4; 15.55–23.8; 24.71–30.00	Wideband	41.9/3.3	3.3, 2.4, 5.2	two metallic rectangular ring resonators, single layer	Radar, satellite communication
[20]	12.94–16.54; 17.54–26	Ultra-wideband	38.9/24.4	4.09, 2.57	three anisotropic stair-shaped resonators, single layer	navigation systems, satellite communication, and imaging systems
[21]	7.48–10.55; 18.47–19.52	Wideband	34.1/5.5	2.94, 18.09	Orthotropic reflector metasurface, single layer	satellite systems, navigation systems, and stealth surfaces
This Method	4.71–5.44; 7.26–9.55; 11.62–12.6; 13.33–13.46; 15.61–15.62	Wideband and ultra-narrowband coexistence	27.2/0.06	6.95, 3.67, 12.35, 103.04, 1561.5	Forked-crossing patch, single layer	Multi-band devices, radars, target locking

## Data Availability

Data will be made available on request.
